# Jujube promotes learning and memory in a rat model by increasing estrogen levels in the blood and nitric oxide and acetylcholine levels in the brain

**DOI:** 10.3892/etm.2013.1063

**Published:** 2013-04-11

**Authors:** BAOLI LI, LU WANG, YONGXIAN LIU, YAHUI CHEN, ZHENGXIANG ZHANG, JING ZHANG

**Affiliations:** Department of Pharmacology, Medical College of Yan’an University, Yan’an, Shaanxi 716000, P.R. China

**Keywords:** jujube, learning and memory, nitric oxide synthase, choline acetyltransferase, cholinesterase

## Abstract

The aim of this study was to observe the effects of jujube on learning and memory in ovariectomized rats. The effects of jujube on learning and memory in ovariectomized rats were observed using the Morris water maze method. The serum follicle-stimulating hormone (FSH), estrogen and luteinizing hormone (LH) levels, and the brain nitric oxide synthase (NOS) and acetylcholinesterase (AChE) levels of the rats were determined. The results indicated that jujube reduced the latency period and increased the number of crossings made by the ovariectomized rats in the Morris water maze test. Jujube also increased the serum estrogen level, reduced the serum FSH and corpus luteum LH levels, increased brain NOS activity and reduced AChE activity. The results indicate that jujube promoted the learning and memory of the ovariectomized rats. This effect may be correlated with the increase in the estrogen level in the blood, and the changes in the nitric oxide and acetylcholine levels in the brain.

## Introduction

Females undergoing the climacteric may exhibit a series of symptoms known as perimenopausal syndrome, which occur due to an imbalance of neuroendocrine regulatory mechanisms caused by ovarian failure and reduced estrogen levels in the body (1,2). Hormone replacement therapy, which is predominantly used for the treatment of perimenopausal ovarian hypofunction, has limitations in clinical application due to various contraindications and adverse reactions (3). Thus, novel natural medicines with estrogen-like activity are necessary. An important consideration for the action mechanism of such medicines is how they may delay ovarian aging.

Certain molecules from natural sources, including oleic acid (4,5), linoleic acid (6), and linolenic acid (7), have been found to be helpful for improving learning and memory. Oleic acid and linoleic acid react to produce γ-linolenic, dihomo-γ-linolenic and arachidonic acid, as well as prostaglandins and leukotrienes (8). α-linolenic acid, after it enters the body, exists mainly in the forms of eicosapentaenoic acid (EPA) and docosahexaenoic acid (DHA) (9). Arachidonic acid, EPA and DHA are essential for brain development, and for learning and memory functions. This is due to their ability to upregulate the release of acetylcholine (ACh) and participate in long-term potentiation (LTP) and the regulation of synaptic plasticity, thus promoting the learning capability of the subject animals (10–13). EPA raises the intracellular Ca^2+^ concentration and thus enhances LTP, as well as memory formation and consolidation (14).

We recently examined the effects of Ziziphi Spinosae and Fructus Gardeniae, two herbs commonly used in traditional Chinese medicine, on the learning and memory of mice in a Morris water maze test (15). It was observed that the compound extracts of Ziziphi Spinosae and Fructus Gardeniae had a synergistic effect on the learning and memory of mice (15). In the current study, the effect of jujube on the learning and memory of ovariectomized rats was investigated using the Morris water maze test. The related anti-declining mechanisms of learning and memory were also explored.

## Materials and methods

### Animals

Sixty healthy three-month-old female Sprague Dawley^®^ (SD) rats (body weight 280±20 g) were provided by the Laboratory Animal Center, College of Medicine, Xi’An Jiaotong University (license no. Shaan 08-005; Xi’an, China).

### Compounds

Raw materials containing jujube were purchased from Xuhuang Botanical Science and Technology Co., Ltd. (cat. no. XHS-080107; Xi’an, China). Diethylstilbestrol was obtained from Shijiazhuang City Xiehe Pharmaceutical Co., Ltd. (lot no: H13021146; Shijiazhuang, China).

### Devices and reagents

Devices used in the study included a KDC-160 high-speed refrigerated centrifuge (Keda chuangxin Company, Hefei, China); Sanyo Ultra-Low −135°C freezers (Sanyo Biomedical Solutions, Secaucus, NJ, USA); an MT-200 Morris water-maze tracking system (Chengdu Technology and Market Co., Ltd., Chengdu, China); an electronic balance (Beijing Sartorius Instrument System Co., Ltd., Beijing, China); a system microscope (Olympus BX41; Olympus Corporation, Prince Park Tower Tokyo, Japan) and a TU-1800PC UV-VIS spectrophotometer (Beijing Persee General Instrument Co. Ltd., Beijing, China). A Roche COBAS E411 fully-automated chemiluminescence immunology analyzer (Roche Diagnostics, Basel, Switzerland) was used to measure the levels of estrogen, follicle-stimulating hormone (FSH) and corpus luteum luteinizing hormone (LH) via electrochemiluminescence immunoassays. The diagnostic reagents, standards and quality controls were also provided by Roche Diagnostics. Reagent kits used included a nitric oxide synthase (NOS) assay kit (cat. no. 20080913), a choline acetyltransferase (ChAT) enzyme-linked immunosorbent assay (ELISA) kit (cat. no. 20080721) and a acetylcholinesterase test kit (cat. no. 20080515), all of which were purchased from Nanjing Jiancheng Bioengineering Institute (Nanjing, China).

### Experimental grouping

Sixty healthy female SD rats were subjected to intraperitoneal anesthesia with 3% pentobarbital sodium (1 ml/kg), and then to a bilateral ovarian resection under aseptic conditions. Following surgery, an intramuscular injection of 400,000 units penicillin was immediately administered to each rat to protect against infection. The rats were randomly divided into six groups, three of which were treated with high, medium and low levels of jujube, respectively, one of which was treated with diethylstilbestrol and one of which was the model group. The final group was the sham surgery group, in which the rats were subjected to the same anesthetic and surgical treatments as the ovariectomized group, except that equal volumes of fat tissue surrounding the ovaries were removed instead of the ovaries. All rats were subject to an exfoliative cytoscopy of the vagina two weeks post-surgery. Unlike those in the sham surgery group, the rats in the ovariectomized group had alterations in their estrous cycles, which confirmed the success of the model construction.

Drugs were administered to the rats via 3 ml saline gavage from four weeks after the surgery. The high-, medium- and low -dosage groups were treated with 4.50, 1.80 and 0.72 g/kg jujube, respectively. The diethylstilbestrol group was treated with 0.02 mg/kg diethylstilbestrol, while the model and sham surgery groups were treated with equal volumes of physiological saline.

The compounds were administered to the rats for 12 consecutive weeks. Each day between days 121 and 125, the rats were trained 1 h after receiving the drugs, and their learning and memory performance was evaluated. The rats were treated with the compounds for the final time on day 126, after being maintained in a fasted state for 12 h, and were anesthetized 1 h later for sample tissue collection.

### Blood tests

The rats were subject to intraperitoneal anesthesia with 3% pentobarbital sodium (1 ml/kg) after being weighed, and their blood was collected via duct drainage from the common carotid artery. The blood serum was isolated and stored in a freezer at −80°C for sex hormone determination at a later date.

### Determination of hormone levels in brain tissues

The animals were put on ice following the collection of blood from the common carotid artery, and the cerebral cortex was immediately isolated. The cerebrum (without the cerebellum attached) was weighed precisely, homogenized in an ultrasonic homogenizer and combined with 9-fold of its volume of ice-cold physiological saline solution to prepare a 10% tissue homogenate for testing. The colorimetric determinations of NOS, ChAT and acetylcholinesterase (AChE) levels were performed in accordance with the instructions provided with the testing kits.

### Learning and memory tests

The Morris water maze is widely used for the assessment of learning and memory in rats, and usually involves two experiments: i) the object location and navigation experiment, where latency is used to measure the learning performance of the rat; and ii) the spatial probe experiment, where the number of times the rat passes the original location of the platform is used to measure the memory retention capability of the rat. The rats were trained for the Morris water maze test 1 h after taking the compounds, between 9:00 and 12:00 a.m. The water temperature was set at 22±2°C. The pool was divided into four quadrants. In each quadrant, a platform measuring 12 cm in diameter and 20 cm in height was placed 33 cm from the wall of the pool and 2 cm below the water surface. A camera was installed above the maze to record the real-time paths of the rats.

**Figure 1:**
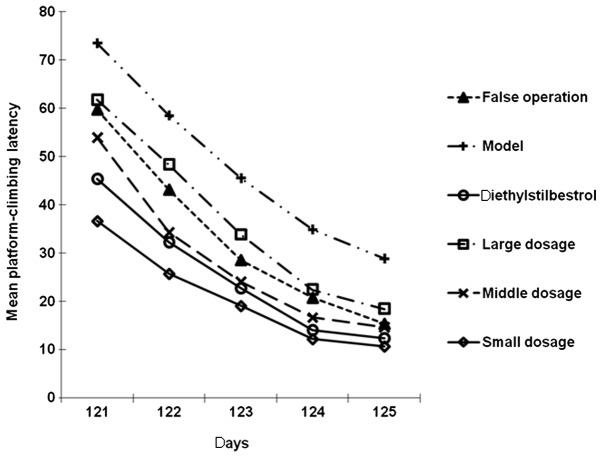
Influence of jujube on the mean platform-climbing latency of ovariectomized rats using the Morris water maze. Rats in three of the groups were treated with jujube (4.50, 1.80, and 0.72 g/kg in the high-, medium- and low-dosage groups, respectively). The diethylstilbestrol group was treated with 0.02 mg/kg diethylstilbestrol, while the model and sham surgery groups were treated with equal volumes of physiological saline. In the sham surgery group, the rats were subjected to the same anesthetic and surgical treatments as the other groups, except that equal volumes of fat tissue surrounding the ovaries were removed instead of the ovaries. Rats were placed on the platform for 20 sec to enable them to adapt, and were then placed into the pool with their backs against a platform in a different quadrant. The time taken by the rats to find the platform was recorded as latency.

For the object location and navigation tests, each rat was placed on a platform for 20 sec to enable it to adapt, and was then placed into the pool with its back against a platform in a different quadrant. The time taken by the rat to find the platform was recorded as latency (Fig. 1). If the rat found the platform, it was kept there for 15 sec. Rats that were not able to find the platform by themselves within 2 min were guided to the platform.

For the spatial probe test, the platform was removed and the rat was placed in the water at the central point of the quadrant, opposite to the platform location. The number of times the rat passed the platform within 120 sec was recorded, as was the path it took. The experiments were repeated four times on each of five consecutive days.

### Statistical analyses

All values are presented in the form of mean ± standard deviation. Using the statistical analysis software SPSS 19.0 (SPSS, Inc., Chicago, IL, USA), the data in the water maze test were analyzed using the multivariate general linear model (GLM). The pairwise comparison between different times for each group was made using the least significant difference (LSD) t-test in the univariate analysis of variance (ANOVA). Pairwise comparisons for the remaining data were made using intergroup LSD or Tamhane t- tests in a univariate GLM. P<0.05 was considered to indicate a statistically significant difference.

## Results

### Jujube promotes learning and memory in the rat model

To determine the effect of jujube on learning and memory, an ovariectomized rat model was established. As shown in Table I and Fig. 1, the model group of ovariectomized female rats demonstrated longer latency and passed the platform position fewer times in the water maze tests than the sham surgery group. However, the jujube-treated groups demonstrated shortened latency and passed the platform a greater number of times than the model group (the negative control group), with statistically significant differences. The shortening of latency with increasing days of training was also statistically significant in all three jujube-treated groups. As the positive control, the diethylstilbestrol group exhibited similar effects to the jujube group (Table I).

The model group of ovariectomized female rats demonstrated reduced levels of estradiol (E_2_), and increased levels of FSH and LH, compared with the sham surgery group. The jujube-treated groups, however, demonstrated increased levels of E_2_ and reduced levels of FSH and LH, compared with the model group. The diethylstilbestrol group exhibited similar hormone levels to the jujube groups (Table II). These results suggested that jujube promotes learning and memory in ovariectomized rats.

### Jujube increases the estrogen level in the blood, and nitric oxide (NO) and ACh levels in the brains of the rat models

To determine whether jujube affected the estrogen levels in the blood or the NO and ACh levels in the brains of the rat models, estradiol levels and indicators of NO and ACh levels were measured. As shown in Table III, the model group of ovariectomized female rats demonstrated lower intracerebral NOS and ChAT activity, and higher AChE activity than the sham surgery group; whereas the jujube groups exhibited enhanced intracerebral NOS and ChAT activities and reduced AChE activity compared with the model group. These results were statistically significant. The diethylstilbestrol group demonstrated a similar effect to the jujube groups (Table III). These results suggest that the role of jujube in the promotion of learning ability and memory in the rat models may correlate with the increased estrogen levels in the blood and increased NO and ACh levels in the brain. In addition, estrogen improved levels of NO and Ach in the brain.

## Discussion

Jujube significantly reduced the latency and increased the number of times the rats passed the platform location in the Morris water maze tests, which indicated that jujube enhanced the learning and memory performance of the ovariectomized rats. The effect of jujube may have been related to its function in significantly raising the estradiol level, decreasing the FSH and luteum LH levels, enhancing intracerebral NOS and ChAT activity and decreasing the AChE activity in the ovariectomized rats.

Previous studies (16) have demonstrated that in the process of LTP, NO diffuses from the postsynaptic to the presynaptic terminal, increases the release of glutamic acid, and thus enhances synaptic transmission. Insufficient levels of NO due to a lack of NOS may impede the formation of LTP and the establishment of synaptic plasticity, and thus influence the acquisition of normal learning and memory behaviors. Other studies (17,18) have also demonstrated that the synthesis of NO is reduced in ovariectomized rats as a result of reduced neuronal NOS activity, which is due to lower levels of endogenous estrogen. However, hormone replacement therapy is able to promote the expression of NOS and thus enhance NO synthesis. A second potential target, intracerebral ACh, is an important medium for the execution and maintenance of the advanced functions of the nervous system, and has a dosage-dependent effect on the enhancement of learning and memory (19). Estrogen is capable of increasing intracerebral ACh levels via the reactivation of ChAT (the rate-limiting synthetase of ACh) and the inhibition of AChE, and thus enhancing learning and memory (20). In the present experiment, jujube may have increased the intracerebral levels of NO and ACh by increasing the level of estrogen, or by functioning as a phytoestrogen, thus leading to the enhanced learning and memory capability of the ovariectomized rats.

The mechanism by which jujube regulates estrogen levels in the human body remains unclear. One possibility is that it is transformed into estrogen via a biochemical conversion or by one of the metabolic processes that occur in the human body. The other possibility is that jujube is able to promote the secretion of estrogen by the ovaries and other glands. It is potentially useful in clinical applications using phytoestrogens for the prevention of symptoms caused by reduced levels of estrogen and may reduce or avoid the side-effects of hormone replacement therapy.

## Figures and Tables

**Table I:** Effects of jujube on the platform-climbing latency and crossing number in the Morris water maze test of the ovariectomized rats.

Group	n	Latency (sec)					Crossing no. in 2 min
		D121	D122	D123	D124	D125	
Sham surgery	10	59.75±38.23	43.15±41.98	28.55±37.78	20.74±20.93	15.35±14.14	2.4±1.2
Model	12	73.50±41.13	58.52±39.45	45.54±33.07	34.91±28.27	28.89±22.14	1.6±1.4
Diethylstilbestrol	9	45.39±45.63	32.27±32.65	22.78±25.49	14.11±10.07^a^	12.37±9.05^a^	2.8±1.6
High dosage	7	61.78±49.75	48.35±46.03	33.84±35.90	22.47±20.26	18.45±16.17	1.8±1.7
Medium dosage	7	54.00±35.75	34.31±33.81	24.10±34.77	16.63±15.04	14.63±14.14	2.5±1.4
Low dosage	8	36.66±32.82^a^	25.73±20.04^a^	19.06±15.13^a^	12.19±8.27^a^	10.68±8.40^a^	3.5±2.3^a^

a P<0.05 vs. the model group. D, day. Values presented are mean ± standard deviation.

**Table II:** Effects of jujube on the sex hormone concentrations of the ovariectomized rats.

Group	n	E_2_ (*μ*M)	FSH (IU/l)	LH (IU/l)
Sham surgery	10	120.36±32.60^a^	0.25±0.07^b^	0.27±0.08^a^
Model	12	77.55±17.64	0.35±0.16	0.44±0.17
Diethylstilbestrol	9	130.36±54.04^a^	0.06±0.04^a^	0.20±0.07^a^
High dosage	7	82.73±20.09	0.27±0.06	0.34±0.10
Medium dosage	7	91.31±19.91	0.26±0.07	0.32±0.07^b^
Low dosage	8	129.80±22.35^a^	0.22±0.07^b^	0.24±0.06^a^

E_2_, estradiol; FSH, follicle-stimulating hormone; LH, luteinizing hormone.

a P<0.01 and

b P<0.05 vs. the model group. Values presented are mean ± standard deviation.

**Table III:** Effects of jujube on the brain NOS, ChAT, and AChE activities of ovariectomized rats.

Group	n	NOS (*μ*mol/mg protein)	ChAT (IU/g)	AChE (*μ*mol/mg protein)
Sham surgery	10	7.45±3.48^b^	225.59±57.63^b^	0.60±0.24^b^
Model	12	4.53±2.87	183.24±31.01	0.87±0.35
Diethylstilbestrol	9	8.16±4.01^b^	276.31±99.36^a^	0.47±0.23^a^
Large dosage	7	5.34±3.37	201.24±46.28	0.78±0.31
Medium dosage	7	6.82±3.14	237.77±62.54^b^	0.58±0.22^b^
Small dosage	8	8.76±4.32^a^	256.31±78.05^a^	0.41±0.26^a^

NOS, nitric oxide synthase; ChAT, choline acetyltransferase; AChE, acetylcholinesterase.

a P<0.01 and

b P<0.05 vs. model group. Values presented are mean ± standard deviation.
